# Piscidin-1 Induces Apoptosis via Mitochondrial Reactive Oxygen Species-Regulated Mitochondrial Dysfunction in Human Osteosarcoma Cells

**DOI:** 10.1038/s41598-020-61876-5

**Published:** 2020-03-19

**Authors:** Meng-Hsuan Cheng, Chieh-Yu Pan, Nan-Fu Chen, San-Nan Yang, Shuchen Hsieh, Zhi-Hong Wen, Wu-Fu Chen, Jin-Wei Wang, Wen-Hsien Lu, Hsiao-Mei Kuo

**Affiliations:** 1 Division of Pulmonary and Critical Care Medicine, Department of Internal Medicine, Kaohsiung Medical University Hospital, Kaohsiung, 80756 Taiwan; 2 School of Medicine, College of Medicine, Kaohsiung Medical University, Kaohsiung, 80708 Taiwan; 3 Department of Respiratory Therapy, College of Medicine, Kaohsiung Medical University, Kaohsiung, 80708 Taiwan; 4 Department and Graduate Institute of Aquaculture, National Kaohsiung University of Science and Technology, Kaohsiung, 81101 Taiwan; 5 Division of Neurosurgery, Department of Surgery, Kaohsiung Armed Forces General Hospital, Kaohsiung, 80284 Taiwan; 6 Department of Neurological Surgery, Tri-Service General Hospital, National Defense Medical Center, Taipei, 11490 Taiwan; 7 Department of Internal Medicine, E-DA Hospital and College of Medicine, I-SHOU University, Kaohsiung, 84001 Taiwan; 8 Department of Chemistry, National Sun Yat-sen University, Kaohsiung, 80424 Taiwan; 9 Department of Marine Biotechnology and Resources, National Sun Yat-sen University, Kaohsiung, 80424 Taiwan; 10 Doctoral Degree Program in Marine Biotechnology, Academia Sinica, Taipei, 11529 Taiwan; 11 Department of Neurosurgery, Kaohsiung Chang Gung Memorial Hospital and Chang Gung University College of Medicine, Kaohsiung, 83301 Taiwan; 12 Department of Neurosurgery, Xiamen Chang Gung Hospital, Xiamen, Fujian China; 13 Department of Orthopedic, Kaohsiung Armed Forces General Hospital, Kaohsiung, 80284 Taiwan; 14 Department of Orthopedic, Feng Yuan Hospital of the Ministry of Health, Taichung, 42055 Taiwan; 15 Center for Neuroscience, National Sun Yat-sen University, Kaohsiung, 80424 Taiwan

**Keywords:** Bone cancer, Target identification

## Abstract

Osteosarcoma (OSA) is the most common type of cancer that originates in the bone and usually occurs in young children. OSA patients were treated with neoadjuvant chemotherapy and surgery, and the results were disappointing. Marine antimicrobial peptides (AMPs) have been the focus of antibiotic research because they are resistant to pathogen infection. Piscidin-1 is an AMP from the hybrid striped bass (*Morone saxatilis* × *M. chrysops*) and has approximately 22 amino acids. Research has shown that piscidin-1 can inhibit bacterial infections and has antinociception and anti-cancer properties; however, the regulatory effects of piscidin-1 on mitochondrial dysfunction in cancer cells are still unknown. We aimed to identify the effects of piscidin-1 on mitochondrial reactive oxygen species (mtROS) and apoptosis in OSA cells. Our analyses indicated that piscidin-1 has more cytotoxic effects against OSA cells than against lung and ovarian cancer cells; however, it has no effect on non-cancer cells. Piscidin-1 induces apoptosis in OSA cells, regulates mtROS, reduces mitochondrial antioxidant manganese superoxide dismutase and mitochondrial transmembrane potential, and decreases adenosine 5′-triphosphate production, thus leading to mitochondrial dysfunction and apoptosis. The mitochondrial antioxidant, mitoTempo, reduces the apoptosis induced by piscidin-1. Results suggest that piscidin-1 has potential for use in OSA treatment.

## Introduction

When immature bones in the body become cancerous, it is called osteosarcoma (OSA), which is the most common type of bone cancer^1^, and is found at the end of long bones, usually around the knee^2^. Clinically diagnosed OSA patients are usually younger than 25 years of age and the cause of OSA is unclear^3^. Current treatments for OSA include neo-adjuvant chemotherapy, followed by surgery and post-operative treatment^4^. The 5-year survival rate for patients with OSA is 60–70%^5^, and the survival rate of patients with metastatic disease (lungs and distal bones) is approximately 15–25%^6,7^. While large clinical trials have repeated attempts to dose-enhance and add chemotherapeutic agents to enhance effectiveness of treatment, the survival rate of OSA remained unchanged for the past thirty years, thus, finding or developing new drugs being the only way to increase the survival rate.

When cells commit suicide, it produces an apoptosis phenomenon, which is sometimes called programmed cell death^8^. In fact, the process of apoptosis follows a controlled, predictable procedure. Mitochondrial reactive oxygen species (mtROS)-induced oxidative stress are capable of causing rapid depolarization of the inner mitochondrial transmembrane potential (MTP) and subsequent disruption of oxidative phosphorylation (OXPHOS). Overproduction of cellular ROS could cause damage to DNA, RNA, proteins, and cell cycle progression, which subsequently lead to apoptotic cell death^9,10^. ROS are the inevitable byproducts of sites on the OXPHOS in the electron transport chain (ETC)^9,11^. In many human diseases, such as cancer, inflammation, Alzheimer, Parkinson’s, and Huntington disease, ROS changes can be observed^12–14^. Recent studies have shown that high concentrations of ROS, as produced from exposure to many chemotherapeutic drugs, can generate a cytotoxic effect and induce apoptosis of cancer cells via disruption of the mitochondrial membrane and function^15,16^. The researches focusing on the effects of ROS generators on the mitochondrial function had been greatly improved by the use of Seahorse XF24 Extracellular Flux Technology and Analyzer, which allows researchers to accurately quantify mitochondrial function and respiration in intact cells^17,18^. Mitochondrial fission and fusion are essential processes that require many specific proteins, including the mechanical enzymes, which physically alter the mitochondrial membrane, and adapter proteins, which modulate the interaction of these mechanical proteins with organelles. The fusion proteins, including mitofusins (MFN1, MFN2) and optic atrophy (OPA1), as well as fission proteins, including mitochondrial fission 1 (FIS1) and dynamin-1-like (DRP1), play an important role in the dynamic changes of the mitochondria^19^, and although incompletely understood, mitochondrial morphological changes appear to be involved in several activities critical to cell health and tumor cells^20^. Based on cellular process involvement, mitochondrial dynamic imbalance may be responsible for the mitochondrial dysfunction of many diseases. In this situation, the unbalance between mitochondrial fission and fusion, as caused by oxidative stress, affects the metabolism and function of the mitochondria, and thus, may be related to the occurrence and development of various human diseases, including neurodegenerative diseases, cardiac metabolic diseases, and cancer^21^. To prevent cellular damage and apoptosis caused by excessive levels of ROS, cells contain antioxidants which act as scavengers of mitochondrial ROS^22^.

Piscidin-1, a natural compound isolated from the mast cells of the hybrid striped bass, is one of the piscidins family, and contains antimicrobial cationic peptides^23,24^. Piscidin-1 peptide was isolated using a previously described method^25^, and was modified to include a further purification step cation-exchange chromatography and reverse-phase high-pressure liquid chromatography using continuous acid-urea polyacrylamide-gel electrophoresis^26^. Previous research shows that piscidin-1 can suppress bacterial proliferation^27^ and inhibit the migration of fibrosarcoma cells^28^. Regarding other cancers, piscidin-1’s effects and the associated mechanism are not very clear. This study aims to investigate the inhibitory abilities of piscidin-1 on various cancer cells, as well as apoptosis-induced by mitochondrial oxidative stress and oxidative phosphorylation.

## Results

### Influence of Piscidin-1 on the cell viability of various cancer and noncancer cells and morphological changes in OSA cells

**Figure 1:**
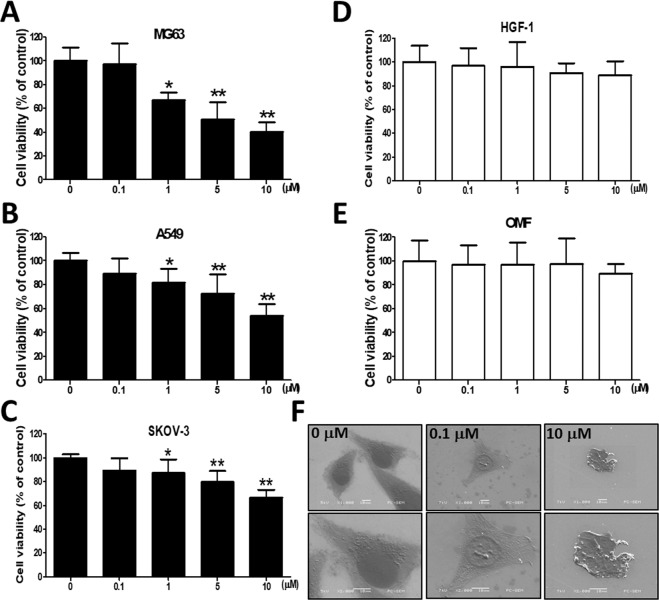
Effect of piscidin-1 on the viability of cancer and noncancer cells and induced morphologic changes in MG63 cells. Piscidin-1 peptide reduced the viability of the following three cancer cell lines: (**A**) human OSA cells (MG63), (**B**) human lung adenocarcinoma (A549) cells, and (**C**) human ovarian cancer (SKOV-3) cells, which were treated with 0, 0.1, 1, 5, or 10 μM piscidin-1 for 24 h and assayed using MTT staining to measure cell viability. Piscidin-1 did not obviously influence the cell viability of two noncancer cell lines. (**D**) Human primary gingival fibroblast cells (HGF-1) cells and (**E**) oral mucosal fibroblast (OMF) cells were treated with the 0, 0.1, 1, 5, or 10 μM piscidin-1 for 24 h and assayed using MTT to measure the changes in cell viability. Cell viability is expressed as a percentage of the untreated control cells (0 μM). The results are expressed as the mean ± SEM of three independent experiments. Significance was determined using Student’s *t-*test. *p < 0.05; **p < 0.01. (**F**) MG63 cells were treated with 0, 0.1, and 10 μM piscidin-1 for 24 h, and images were taken with a scanning electron microscope. The photograph represented 200X (upper) and 400X (lower) magnification.

We first determined the cytotoxicity of treatment with piscidin-1 for 24 h on various cancer and noncancer cell lines by using 3-(4,5-dimethylthiazol-2-yl)-2,5 -diphenyltetrazolium bromide (MTT) staining, and the results showed that the effects of the treatment were dose-dependent. At piscidin-1 concentrations of 1, 5, and 10 μM, the viability of the human OSA cells (MG63 cell line) decreased to 66.99 ± 6.69%, 50.86 ± 14.44%, and 40.15 ± 8.32% of that in the control, respectively (100 ± 11.36%, 0 μM piscidin-1) (Fig. 1A). The similar concentration dose-dependent effect could be observed in piscidin-1-treated OSA 143B cells for 24 h (Fig. S2A). Piscidin-1 increased cytotoxicity in MG63 and 143B cells in a dose-dependent and time-dependent manner after 48 and 72 h treatment (Fig. S1). At piscidin-1 concentrations of 1, 5, and 10 μM, the viability of human lung adenocarcinoma cells (A549 cell line) was clearly reduced to 81.84 ± 11.63%, 72.39 ± 16.40%, and 53.79 ± 10.19% of that in the control, respectively (0 μM, 100 ± 6.52%) (Fig. 1B), whereas the viability of human ovarian cancer cells (SKOV-3 cell line) was also substantially reduced to 87.12 ± 11.46%, 79.61 ± 9.26%, and 66.34 ± 6.79% of that in the control, respectively (100 ± 2.70%, 0 μM piscidin-1) (Fig. 1C); however, piscidin-1 did not exhibit obvious cytotoxic effects on nontumor cells, such as human primary gingival fibroblast cells (HGF-1 cell line) (Fig. 1D) and human oral mucosal fibroblast cells (OMF cell line) (Fig. 1E). The scanning electron microscope micrographs of MG63 cells were observed after treatment with of 0.1 and 10 μM piscidin-1 for 24 h. Apoptosis is the process through which a cell undergoes programmed death. At a magnification of 200X, the structure of the MG63 cells become spherical as their cytoskeletons are digested in 10 μM piscidin-1; at a magnification of 400X, cell shrinkage representing apoptosis is observed (Fig. 1F). These results suggest that although piscidin-1 can inhibit cancer cell viability, it has no influence on noncaner cells.

### Influence of Piscidin-1 on OSA cells apoptosis

**Figure 2:**
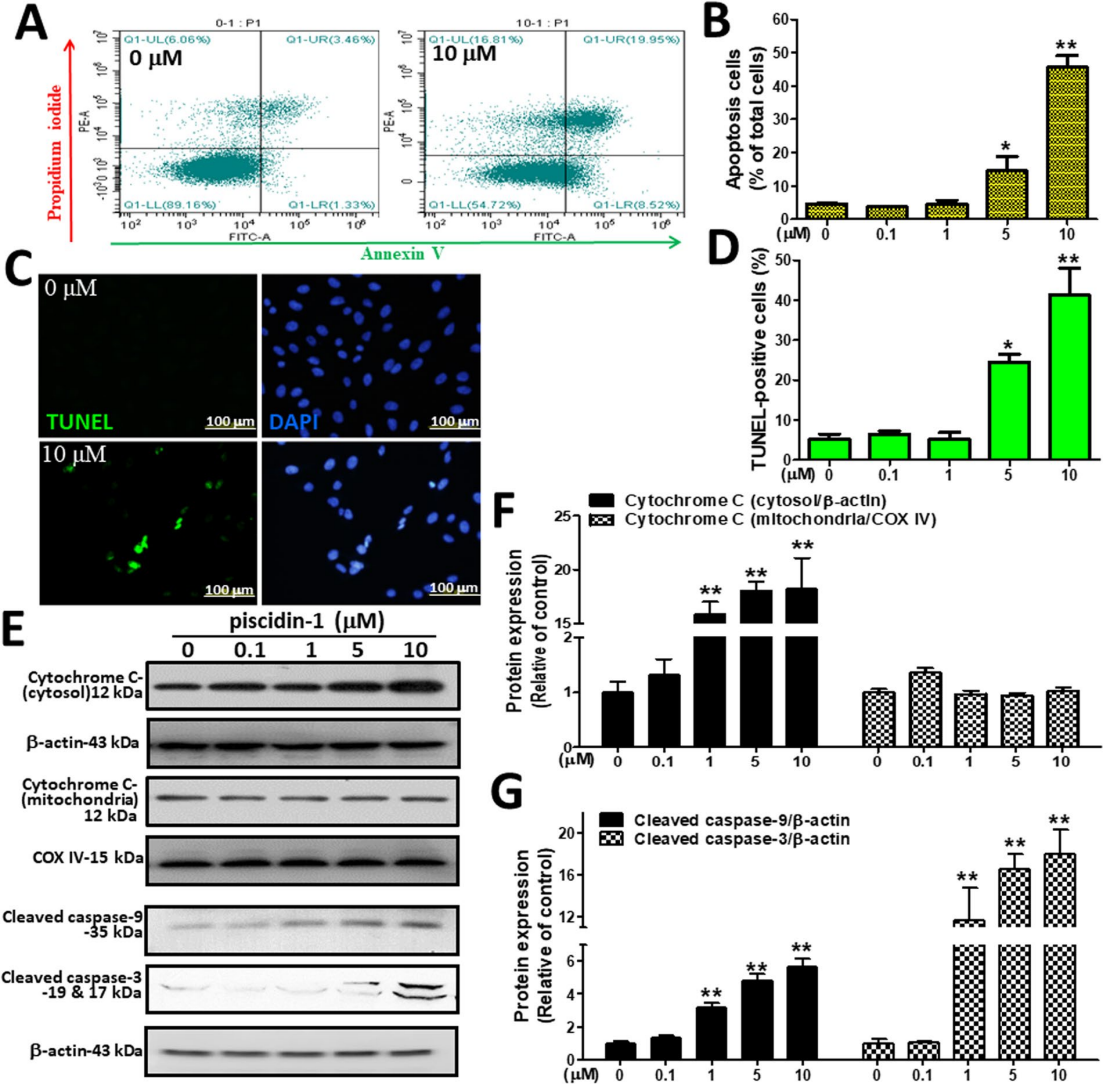
Piscidin-1 induces the apoptosis pathway in the osteosarcoma (OSA) cell line (MG63). (**A**) Apoptosis was determined using annexin V–FITC/PI staining and of the MG63 cells treated with 10 μM piscidin-1 for 24 h. The dot-plot quadrant diagram reflects the annexin V–FITC (x-axis; green) and PI (y-axis; red) in the MG63 cells. (**B**) The percentages of apoptotic cells (lower right quadrant) and dead cells (upper right quadrant) in the MG63 cells treated with the 0, 0.1, 1, 5, and 10 μM piscidin-1 for 24 h were examined using flow cytometry. The apoptotic MG63 cells increased as the concentrations of piscidin-1 increased. Total cells = 20,000; values are the mean ± SEM of three independent experiments. (**C**) Immunofluorescence shows apoptotic bodies in the MG63 cells marked by the TUNEL (green) assay after treatment with the 10 μM piscidin-1 for 24 h. DAPI staining was used to observe cell DNA/nuclei (blue) and was visualized under a laser confocal microscope (200X). (**D**) Statistical analyses of the percentage of TUNEL-positive cells; the values are the mean ± SEM of three independent experiments. (**E**) Protein levels of cytosolic and mitochondrial cyt *c* after various concentrations of piscidin-1 treatment for 24 h. Whole cell lysate proteins were loaded for Western blot analysis by using cleaved caspase-9, cleaved caspase-3, cyt *c*, and β-actin. β-actin and COX IV were used as the cytosol and mitochondria internal controls, respectively. The groupings were cropped from different gels subjected to identical conditions. Full blots are shown in the Supplementary Information, Fig. S4. Cytosol cytochrome *c*, mitochondrial cytochrome *c* (**F**), and protein levels of cleaved caspase-9 and cleaved caspase-3 (**G**) were quantified and normalized to that of β-actin and COX IV and were expressed as fold changes. Significance was determined using Student’s *t-*test. *p < 0.05; **p < 0.01.

To further elucidate the link between the OSA cell apoptosis induced by piscidin-1, we conducted an *in vitro* proof-of-principle study. First, we used annexin V–FITC (green) and propidium iodide (PI)-red staining with flow cytometry dot-plot diagrams to analyze the apoptosis induced by piscidin-1. Figure 2A shows the typical shift between apoptotic cells (annexin V+/PI−) and dead cells (annexin V+/PI+) from the left quadrant toward the right quadrant in MG63 cells treated with piscidin-1 for 24 h. At 5 and 10 μM piscidin-1, the rates of apoptotic and dead MG63 cells (14.48 ± 4.38% and 45.59 ± 3.61%, respectively) obviously increased compared with those in the control (4.61 ± 0.16%, 0 μM piscidin-1) (Fig. 2B), whereas a similar phenomenon can also be observed in piscidin-1-treated 143 B cells (Fig. S2B,C). Furthermore, a terminal deoxy-nucleotidyl transferase dUTP nick end labeling (TUNEL) assay was conducted *in vitro* to evaluate the apoptotic effect of piscidin-1 and observe the apoptotic cells that exhibited extensive DNA fragmentation during apoptosis^29^. TUNEL staining (green) was exhibited using immunofluorescence and showed nuclear condensation and apoptotic bodies in the MG63 cells after treatment with 10 μM piscidin-1, and all nuclei (blue) were stained with 4′,6-diamidino-2-phenylindole (DAPI) (Fig. 2C). Our data show that there was an increasing trend in TUNEL-positive cells in the groups treated with 5 (19.48 ± 4.38%) and 10 μM (40.59 ± 3.60%) piscidin-1 compared with that in the control (5.11 ± 1.39%, 0 μM piscidin-1) (Fig. 2D). The intrinsic apoptosis pathway is initiated by the disruption of the inner mitochondrial membrane under excessive oxidative stress, thus resulting in the release of cytochrome *c* (cyt *c*) protein^30^, whereas the downstream caspase-3 has been identified as an important executioner of the apoptosis process^31^. After normalization of the protein with β-actin, analysis revealed that the exposure of MG63 cells to different concentrations of piscidin-1 for 24 h increased the expression levels of cytosolic cyt *c*, cleaved caspase-9, and cleaved caspase-3 (Fig. 2E). After protein normalization with cyt *c* oxidase complex IV (COX IV), the mitochondrial cyt *c* was not affected. MG63 cells that were treated with different concentrations of piscidin-1 (i.e., 0, 1, 5, and 10 μM) for 24 h exhibited a rapid accumulate in cytoplasmic cyt *c* protein levels of 1.00 ± 0.33, 15.89 ± 1.93, 18.06 ± 1.50, and 18.20 ± 5.00 in a dose-dependent manner, but mitochondrial cyt *c* was not affected (Fig. 2F). The piscidin-1 treatment of the MG63 cells with 1, 5, and 10 μM obviously increased the protein levels of cleaved caspase-9 in a dose-dependent manner to 3.18 ± 0.50, 4.76 ± 0.73, and 5.67 ± 0.86, respectively, compared with that of the control at 1.00 ± 0.17 (0 μM piscidin-1). The piscidin-1 treatment of the MG63 cells with 1, 5, and 10 μM also led to a dose-dependent increase in the levels of cleaved caspase-3 at 11.6121 ± 6.17, 16.52 ± 2.92, and 28.02 ± 4.62, respectively, compared with that of the control at 1.00 ± 0.43 (0 μM piscidin-1) (Fig. 2G). These observations indicated that piscidin-1-induced apoptosis in OSA cells is through the release of cyt c from the mitochondria and the subsequent activation of caspase-9 and caspase-3.

### Effect of Piscidin-1 on mtROS, mitochondrial transmembrane potential, and mitochondrial antioxidant manganese superoxide dismutase

**Figure 3:**
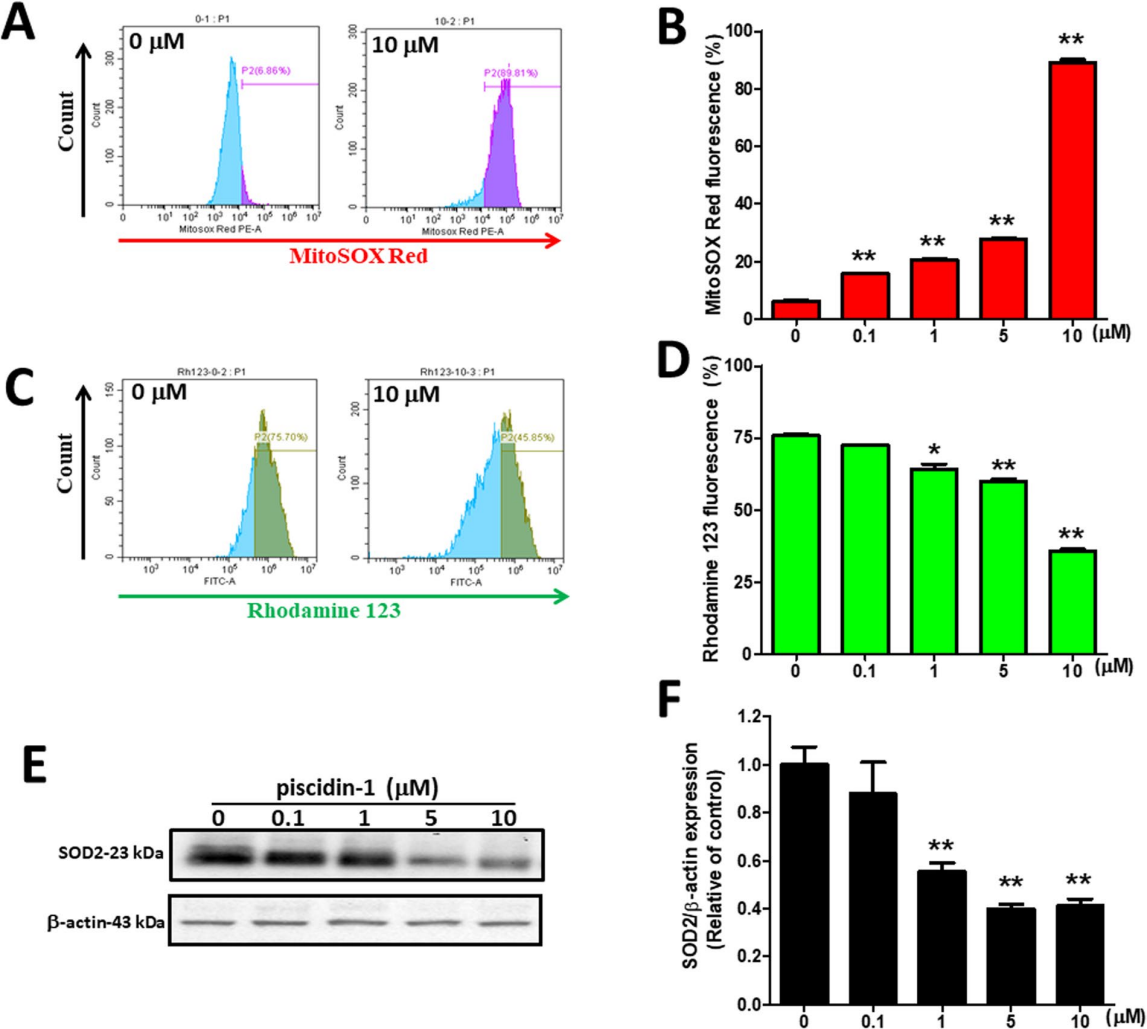
Antimicrobial peptide piscidin-1 induced the production of mtROS, the gradual loss of MTP, and a decrease in mitochondrial antioxidant SOD2 expression in MG63 cells. (**A**) The fluorescent intensity of mtROS was determined using Mitochondrial Superoxide Indicator (MitoSOX Red) (red fluorescence) and was detected by flow cytometry in the MG63 cells treated with different concentrations of piscidin-1 for 24 h. (**B**) Quantification of mtROS accumulation in the mitochondria induced by piscidin-1. The values are the mean ± SEM of three independent experiments. (**C**) MG63 cells treated with 10 μM piscidin-1 for 24 h. MTP depolarization effects were detected using cell-permeable cationic rhodamine 123 and flow cytometry. (**D**) Percentages of MTP-disrupted MG63 cells increased with increasing concentrations of piscidin-1. The quantitative value was obtained by analyzing the gated range of single-parameter histograms from 10^4^–10^7^. (**E**) The cell lysates were used for Western blot analysis with an anti-SOD2 antibody, and β-actin was used as the internal control. The groupings were cropped from different gels subjected to identical conditions. Full blots are shown in the Supplementary Information, Fig. S5. (**F**) SOD2 protein levels were quantified after being normalized with β-actin. The results are expressed as the mean ± SEM of three independent experiments. Significance was determined using Student’s *t*-test; *p < 0.05; **p < 0.01.

ROS is generated mainly by the mitochondria; however, excess ROS production can be the cause of oxidative damage and cell death, followed by loss of mitchondrial transmembrane potential (MTP)^32^. MtROS was detected by flow cytometry using Mitochondrial Superoxide Indicator (MitoSOX Red) stain. MtROS accumulation was enhanced after treatment with 10 μM piscidin-1 for 24 h, and flow cytometry showed a considerable shift to the right in MG63 cells (Fig. 3A). The quantitative results indicated that at concentrations of 0.1, 1, 5, and 10 μM piscidin, mtROS in MG 63 cells clearly increased, in a dose-dependent manner, to 15.75 ± 0.42%, 20.66 ± 0.64%, 27.63 ± 0.77%, and 89.01 ± 2.04%, respectively, compared with that in the control (6.15 ± 0.82%, 0 μM piscidin-1) (Fig. 3B). A similar phenomenon could also be observed in piscidin-1-treated 143 B cells (Fig. S2D,E). A rhodamine 123 probe was used to detect changes in MTP after piscidin-1 treatment. In the MG63 cells treated with 10 μM piscidin-1 for 24 h, MTP was disrupted, and the flow cytometry histogram showed a considerable shift to the left (Fig. 3C). The quantitative results indicated that at concentrations of 1, 5, and 10 μM piscidin-1, the MTP in MG 63 cells apparently decreased to 64.19 ± 2.90%, 59.87 ± 1.44%, and 35.66 ± 1.28%, respectively, compared with that in the control (75.85 ± 0.99%, 0 μM piscidin-1) (Fig. 3D). The same phenomenon was also seen in piscidin-1-treated 143B cells (Fig. S2F,G). Mitochondrial antioxidant manganese superoxide dismutase (SOD2 or MnSOD) is located in the mitochondrial matrix and protects it against excessive oxidative stress^33^. Thereafter, we examined whether antioxidants were involved in the ROS overproduction induced by piscidin-1 in MG63 cells. The results revealed that piscidin-1 treatment downregulated SOD2 protein expression (Fig. 3E) and decreased SOD2 protein levels in the MG63 cells from 1.00 ± 0.13 in the control to 0.55 ± 0.06, 0.40 ± 0.04, and 0.41 ± 0.05 after treatment with 1, 5, and 10 μM piscidin-1, respectively (Fig. 3F). These results suggested that mtROS overproduction, antioxidant (SOD2) reduction, and MTP disruption contribute to the cell apoptosis induced by piscidin-1.

### Effect of Piscidin-1 on oxygen consumption rate in OSA cells

**Figure 4:**
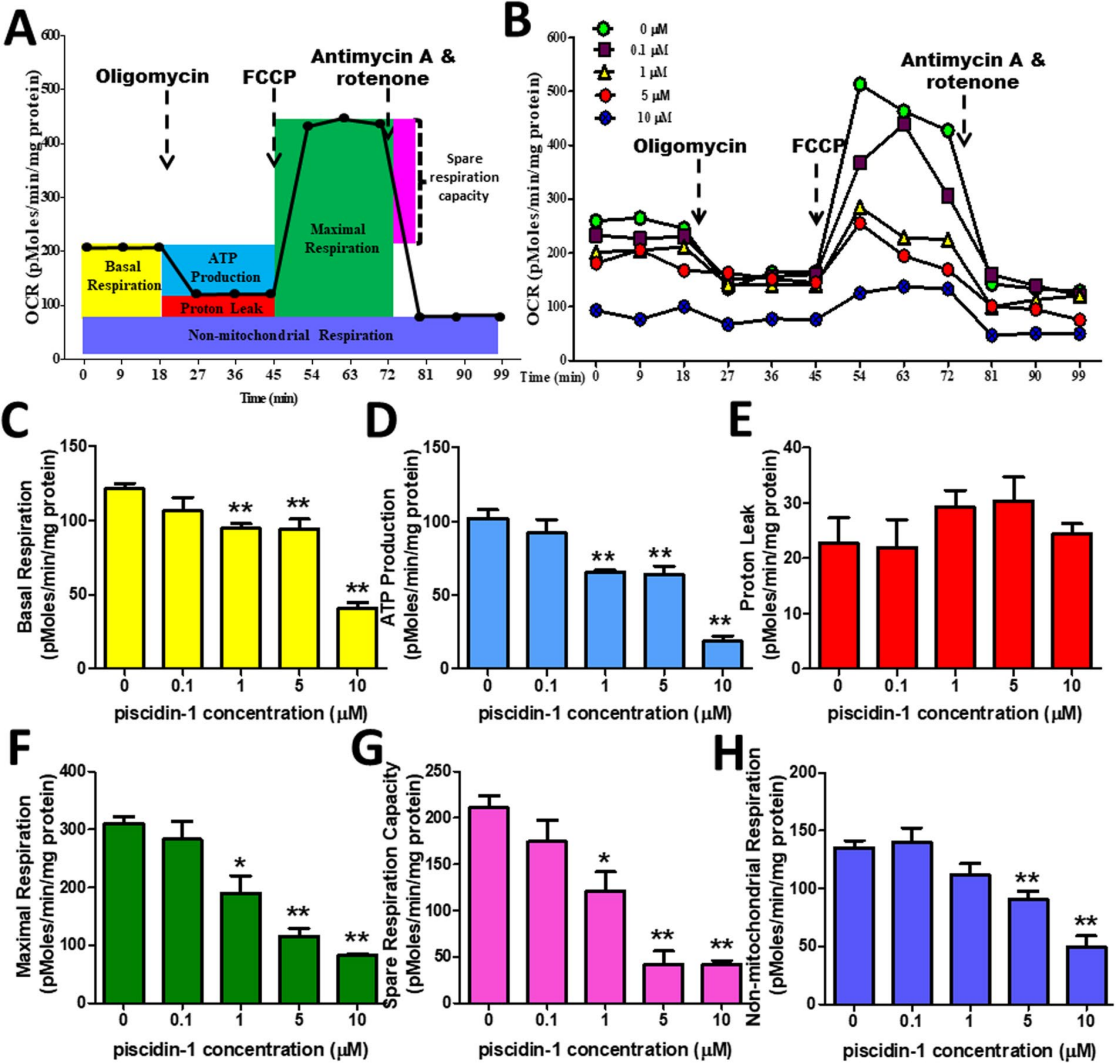
Effect of piscidin-1 on mitochondrial function and respiration in the MG63 cell line. (**A**) The OCR was measured before and after adding pharmacological agents to living cells. Three measurements were taken and averaged to provide reliable data at the start, followed by sequential and continuous injections of oligomycin, FCCP, and antimycin A/rotenone. These analyses measured the basic parameters of basal respiration, ATP production, proton leak, maximum respiration, spare respiratory capacity, and non-mitochondrial respiration. (**B**) OCR value and time-course curve plot. The parameters quantified and analyzed in MG63 cells treated with 0, 0.1, 1, 5, and 10 μM piscidin-1 were the (**C**) basal respiration OCR, (**D**) ATP production (couple respiration), (**E**) proton leak, (**F**) maximal respiration, (**G**) spare respiratory capacity, and (**H**) non-mitochondrial respiration. OCR values were quantified by normalizing the cell protein concentration. The results are expressed as the mean ± SEM of three independent experiments. Significance was determined by Student’s t-test; *p < 0.05; **p < 0.01.

A previous study has reported that oxidative stress causes changes in mitochondrial oxygen consumption in many different types of cancer cells^34^. The mitochondrial oxygen consumption rate (OCR), which is a measure of mitochondrial respiration and mitochondrial activity, was measured in the MG63 and 143B cells treated with piscidin-1 for 24 h. Several mitochondrial respiratory phase profiles were calculated on the basis of the OCR after sequentially and continuously adding oligomycin, 2-[2-[4-(trifluoromethoxy) phenyl] hydrazinylidene]-propanedinitrile (FCCP), and antimycin A/rotenone respiratory inhibitors to inhibit the ETC (Fig. 4A). Treatment with various concentrations of piscidin-1 in MG63 cells decreased OCR (y-axis, pMole/min/mg protein) in a dose-dependent manner (Fig. 4B). As the concentration of piscidin-1 increased, the basal respiration OCR values apparently decreased to 94.53 ± 10.15, 94.02 ± 20.47, and 40.87 ± 10.80 pMole/min/mg protein at concentrations of 1, 5, and 10 μM piscidin-1, respectively, compared with that in the control (121.48 ± 10.57 pMole/min/mg protein, 0 μM piscidin-1) (Fig. 4C). Statistically differences were observed in adenosine 5′-triphosphate (ATP) production, thus indicating that the OCR values of coupled respiration (ATP production) were attenuated to 65.31 ± 4.82, 63.66 ± 17.78, and 18.59 ± 10.45 pMole/min/mg protein in concentrations of 1, 5, and 10 μM piscidin-1, respectively, compared with that in the control (102.13 ± 17.12 pMole/min/mg protein, 0 μM piscidin-1) (Fig. 4D). No obvoius changes were observed in the trend for proton leak (uncoupled respiration) after the same treatment with various concentrations of piscidin-1 for 24 h (Fig. 4E). Adding 1, 5, and 10 μM piscidin-1 obviously reduced the maximum respiration to 190.24 ± 88.63, 115.40 ± 40.13, and 82.86 ± 5.64 pMole/min/mg protein, respectively, compared with that in the control (310.28 ± 35.46 pMole/min/mg protein, 0 μM piscidin-1) (Fig. 4F). The spare respiration capacity also exhibited a dose-dependent reduction from 211.03 ± 38.21 in the control at a concentration of 0 μM piscidin-1 to 121.24 ± 61.06, 42.17 ± 34.29, and 41.99 ± 11.90 pMole/min/mg protein at concentrations of 1, 5, and 10 μM, respectively (Fig. 4G). The non-mitochondrial respiration graph showed that as the concentration of piscidin-1 increased, the non-mitochondrial respiration value or extracelluar acidification rates decreased to 90.88 ± 20.05 and 49.47 ± 28.56 pMole/min/mg protein at 5 and 10 μM piscidin-1, respectively, compared with that in the control (135.25 ± 18.27 pMole/min/mg protein, 0 μM piscidin-1) (Fig. 4H). Piscidin-1 similarly inhibited mitochondrial respiration in 143B cells (Fig. S3). These results suggest that piscidin-1 effectively decreases basal respiration, ATP production, maximum respiration, spare respiratory capacity, and non-mitochondrial respiration in both OSA cells; the proton leak remains unaffected.

### Effects of antimicrobial peptide Piscidin-1 on ETC Complex I–V Proteins and ATP production in MG63 cells

**Figure 5:**
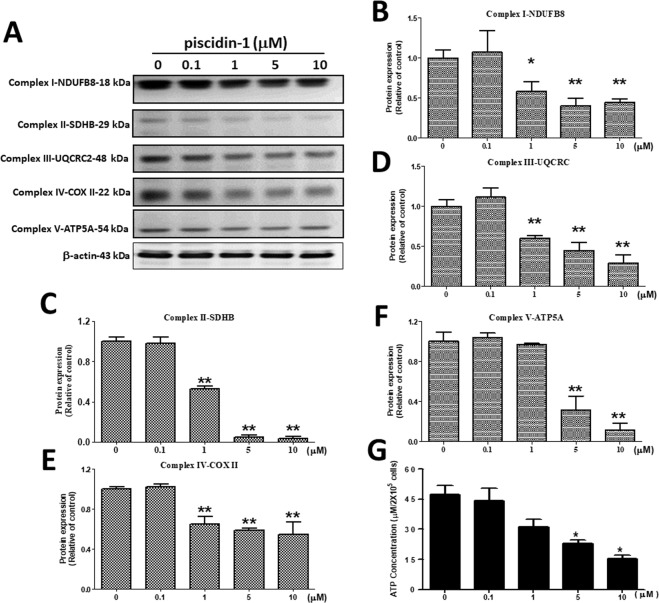
Effect of piscidin-1 on the expression levels of mitochondrial complex I–V protein and ATP production in human MG63 cells. (**A**) Cells treated with 0, 0.1, 1, 5, and 10 μM piscidin-1 for 24 h. The proteins of total cellular lysates were analyzed using Western blot analysis. The profile shows the effects of piscidin-1 treatment on the expression levels of complex I (NDUFB8), complex II (SDHB), complex III (UQCRC2), complex IV (COX II), complex V (ATP5A), and β-actin (as an internal control). The groupings were cropped from different gels subjected to identical conditions. Full blots are shown in the Supplementary Information, Fig. S6. The complex I subunit (**B**), complex II subunit (**C**), complex III subunit (**D**), complex IV subunit (**E**), and complex V subunit (**F**) protein levels were quantified and normalized to the β-actin level. (**G**) The effects of the total ATP production in MG63 cells treated with various concentrations of piscidin-1 for 24 h were measured using an ATP fluorescence kit. The results are expressed as the mean ± SEM of three independent experiments. Significance was determined using Student’s *t-*test; *p < 0.05; **p < 0.01.

The mitochondrial inner membrane contains important ETC organs that act as sites for OXPHOS and ATP synthesis, which is a major mitochondrial function^35^. Western blot analysis was conducted to examine the changes in the protein expressions of the ETC family after piscidin-1 treatment, including complex I (NDUFB8), complex II (SDHB), complex III (UQCRC2), complex IV (COX II), and complex V (ATP5A) proteins. The results showed that the exposure of MG63 cells to different concentrations of piscidin-1 for 24 h substantially reduced the expression levels of mitochondrial ETC complexes I-V proteins (Fig. 5A). Different concentrations of piscidin-1 treatment on MG63 cells for 24 h led to drastically reduced complex I (NADH dehydrogenase) protein levels and was normalized to β-actin protein from 1.00 ± 0.10 (control, 0 μM piscidin-1) to 0.58 ± 0.12, 0.40 ± 0.09, and 0.44 ± 0.04 at 1, 5, and 10 μM piscidin-1 in a dose-dependent manner, respectively (Fig. 5B). The piscidin-1 treatment of MG63 cells for 24 h drastically reduced transmembrane respiratory complex II protein (succinate dehydrogenase) levels from 1.00 ± 0.05 (control, 0 μM piscidin-1) to 0.53 ± 0.03, 0.05 ± 0.02, and 0.03 ± 0.02 at 1, 5, and 10 μM piscidin-1, respectively (Fig. 5C). A similar phenomenon was observed in the mitochondrial complex III (ubiquinol–cytochrome c reductase complex) protein, which was reduced in a dose-dependent manner from 1.00 ± 0.09 (control, 0 μM piscidin-1) to 0.60 ± 0.03, 0.45 ± 0.10, and 0.29 ± 0.10 in cells treated for 24 h with 1, 5, and 10 μM piscidin-1 (Fig. 5D). The expression levels of complex IV (cyt *c* oxidase) protein in the MG63 cells were obviously downregulated after treatment for 24 h with 1 (0.65 ± 0.08), 5 (0.59 ± 0.02), and 10 μM (0.55 ± 0.12) piscidin-1 compared with that in the control at 0 μM piscidin-1 (1.00 ± 0.02) (Fig. 5E). The expression levels of mitochondrial membrane ATP synthase (complex V) protein in the MG63 cells were reduced after treatment for 24 h with 5 (0.3 ± 0.14) and 10 μM (0.11 ± 0.07) piscidin-1 compared with that in the control at 0 μM piscidin-1 (1.00 ± 0.10) (Fig. 5F). The ATP concentrations markedly decreased in the MG63 cells after treatment for 24 h with 5 (22.86 ± 2.58 μM/2 × 10^5^ cells) and 10 μM (15.22 ± 2.60 μM/2 × 10^5^cells) piscidin-1 compared with that in the control at 0 μM piscidin-1 (47.38 ± 6.40 μM/2 × 10^5^cells) (Fig. 5G). These results suggest that piscidin-1 can effectively decrease the expression levels of complexes I, II, III, IV, and V protein and inhibit ATP production in the MG63 cells.

### Effects of Piscidin-1 on mitochondrial dynamic proteins

**Figure 6:**
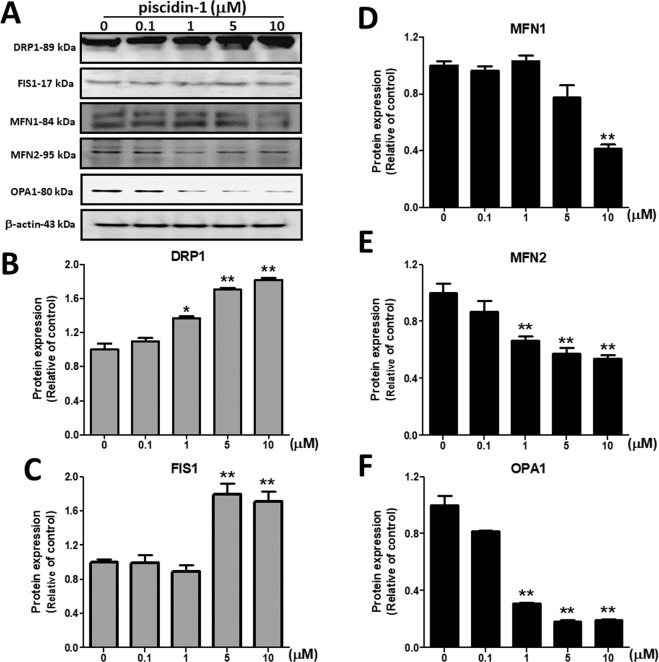
Effects of piscidin-1 on the expression levels of mitochondrial dynamic proteins in MG63 cells. Western blot analysis of mitochondrial fission and fusion proteins in MG63 cells. (**A**) MG63 cells treated with the indicated concentrations of piscidin-1 for 24 h. β-actin used as the internal control. DRP1 (**B**) and FIS1 (**C**) of the fission proteins. MFN1 (**D**), MFN2 (**E**), and OPA1 (**F**) of the fusion proteins. The groupings were cropped from different gels subjected to identical conditions. Full blots are shown in the Supplementary Information, Fig. S7. Data (mean ± SEM) were obtained from three independent experiments. *p < 0.05; **p < 0.01 versus untreated cells.

The mitochondrial fusion proteins were MFN1, MFN2, and OPA1; the mitochondrial fission proteins were DRP1 and FIS1. Our results showed that piscidin-1 treatment for 24 h obviously reduced the expression levels of MFN1, MFN2, and OPA1 fusion proteins but enhanced those of DRP1 and FIS1 fission proteins in MG63 cells (Fig. 6A). Piscidin-1 treatment increased DRP1 protein levels in a dose-dependent manner from 1.00 ± 0.12 in the control (0 μM) to 1.37 ± 0.04, 1.71 ± 0.04, and 1.82 ± 0.04 at 1, 5, and 10 μM piscidin-1, respectively (Fig. 6B). Piscidin-1 treatment also increased the FIS1 protein levels from 1.00 ± 0.05 in the control (0 μM) to 1.79 ± 0.22 and 1.71 ± 0.21 at 5 and 10 μM piscidin-1, respectively (Fig. 6C). Furthermore, our results demonstrated that piscidin-1 reduced the MFN1 protein levels from 1.00 ± 0.05 in the control (0 μM) to 0.41 ± 0.05 at 10 μM piscidin-1 (Fig. 6D). MFN2 protein was also reduced in the cell to 0.66 ± 0.05, 0.56 ± 0.07, and 0.54 ± 0.05 after treatment with 1, 5, and 10 μM piscidin-1 for 24 h, respectively, compared with that in the control (1.00 ± 0.16, 0 μM) (Fig. 6E). Western blot analysis also showed a considerable reduction in OPA1 protein expressions to 0.31 ± 0.01, 0.18 ± 0.01, and 0.19 ± 0.01 in the cells treated for 24 h with 1, 5, and 10 μM piscidin-1 compared with that in the control (1.00 ± 0.11, 0 μM) (Fig. 6F). These results suggested that piscidin-1 can effectively increase the expression level of mitochondrial fission proteins and decrease the expression level of fusion proteins in MG63 cells.

### Pretreatment with mtROS scavengers rescue the mtROS increase, MTP dissipation, and apoptosis induced by Piscidin-1

**Figure 7:**
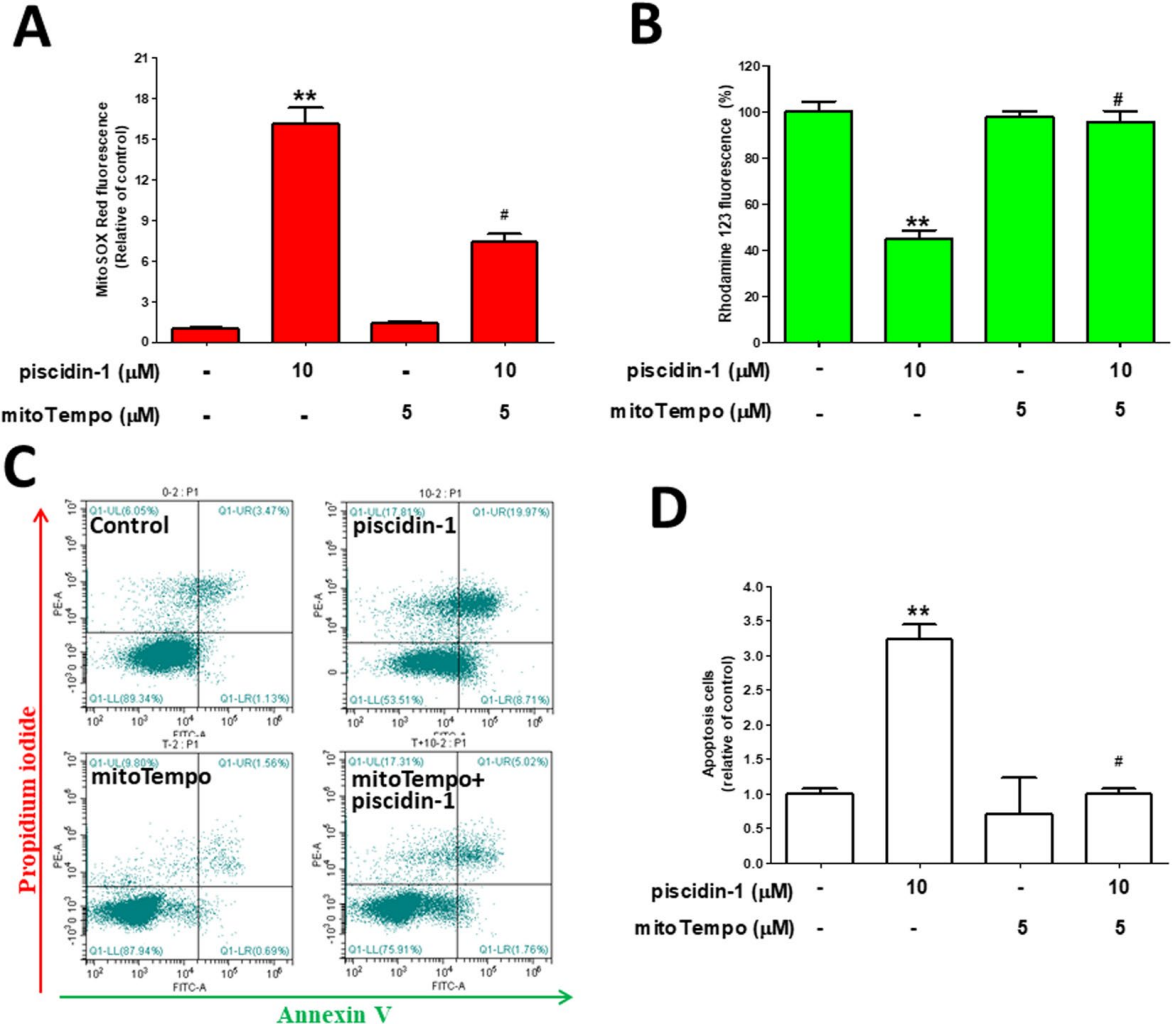
Pretreatment with mitoTempo partially reversed the mtROS accumulation, MTP collapse, and apoptosis induced by piscidin-1 in MG63 cells. (**A**) Histogram of the fold changes after different treatment conditions compared with that in the control. (**B**) Flow cytometry histogram analyses of MTP after treatment with 10 μM piscidin-1, 5 μM MitoTempo, or both for 24 h. The loss of MTP (%) compared to untreated cells. (**C**) Annexin V/PI staining and flow cytometry dot analysis of MG63 cells after incubation with 5 μM mitoTempo for 2 h and treated with or without 10 μM piscidin-1 for an additional 24 h. (**D**) Quantification of apoptosis. The results are expressed as the mean ± SEM of three independent experiments. Significance was determined by Student’s *t-*test. **p < 0.01 versus control; #*p* < 0.05 versus piscidin-1 group.

mitoTempo is a mitochondria-targeting antioxidant and an mtROS scavenger, i.e., it protects the mitochondria from oxidative damage^36^. To determine the effect of mitoTempo on mtROS overproduction, MTP dissipation, and apoptosis induced by piscidin-1, MG63 cells were pretreated with or without 5 μM mitoTempo for 2 h. Thereafter, 10 μM piscidin-1 was added and allowed to react for 24 h. The fluorescence intensity of MitoSOX Red showed a 7.38 ± 0.59-fold change in the mitoTempo and piscidin-1 cotreatment group and a 16.09 ± 1.24-fold change in the piscidin-1-only treatment group compared with that in the dimethyl sulfoxide (DMSO) control group, thus indicating that cotreatment with mitoTempo substantially reduced mtROS production in MG63 cells treated with piscidin-1 (Fig. 7A). The piscidin-1 (10 μM) treatment of MG63 cells for 24 h induced MTP dissipation, but the pretreatment of the cells with mitoTempo effectively rescued this dissipation from an MTP of 44.69 ± 3.87% to an MTP of 95.72 ± 4.67% (Fig. 7B). To evaluate whether mitoTempo attenuates the cell apoptosis induced by piscidin-1, MG63 cells were treated with piscidin-1, mitoTempo, or both for 24 h. The proportion of dead cells (upper right) and apoptotic cells (lower right) were calculated using the gated quadrant dot-plot profile (Fig. 7C). The fold change was 0.99 ± 0.09 in the group treated with both mitoTempo and piscidin-1 and was 3.23 ± 0.22 in the group treated with only piscidin-1 compared with that in the DMSO vehicle control, thus suggesting that there was a considerable reduction in cell apoptosis from cotreatment with mitoTempo (Fig. 7D). Our data indicate that piscidin-1 treatment increased mtROS production, disrupted MTP integrity, and promoted cell apoptosis, whereas pretreatment with mitoTempo sharply suppressed mtROS and cell apoptosis and reduced MTP dissipation.

## Discussion

Piscidin-1 peptide, which was generously donated by the laboratory of Professor Jyh-Yih Chen at Academia Sinica, Jiaushi, Ilan, Taiwan, is one of the AMP series and natural marine compounds isolated from the mast cells of the hybrid striped bass (*Morone saxatilis* × *M. chrysops*). Piscidin-1 is composed of approximately 22 amino acid peptides with amphiphilic α-helical conformation structure and has a small molecular weight of ~2.5 kDa. Previous studies have shown that piscidin-1 can inhibit bacterial proliferation^27^, induce Hela and fibrosarcoma cell apoptosis^28^, and relieve pain^37^. Our results demonstrate that piscidin-1 obviously reduces the cell viability of MG63 cells (IC_50_ = 6.72 ± 2.31 µM) and 143 cells (IC_50_ = 7.15 ± 1.55 µM) compared with that in A549 cells (IC_50_ = 9.93 ± 1.25 µM) and SKOV3 cells (IC_50_ = 13.56 ± 1.84 µM); however, piscidin-1 has no effect on HGF-1 or OMF. The process of apoptosis is generally identified by distinct morphological characteristics (including DNA strand breaks, externalization of phosphatidylserine residues on the cell surface, cell shrinkage, chromosome condensation, and cell apoptotic body formation) and is an energy-dependent mechanism^36^. Most natural marine compounds with anticancer properties have complex action mechanisms, among which the regulations of the apoptosis induction and mitochondrial dysfunction signaling networks play key roles^38^. First, we observed the morphological changes in OSA cells treated with piscidin-1 by using scanning electron microscopy, and the results showed that the structure of the cell cytoskeleton became spherical at low concentrations of the peptide. High concentrations of the peptide induced cell shrinkage, which represents cell apoptosis. The additional examinations using flow cytometric annexin V/PI staining, TUNEL fluorescence assay, and Western blot analysis using antibodies against apoptosis factors (cyt *c*, cleaved caspase-9, and cleaved caspase-3) confirmed that piscidin-1 induces apoptosis. The current study demonstrated that piscidin-1 treatment inhibits OSA cell growth at low concentrations, with an IC_50_ of ~6–7 µM. Lin *et al*. reported that piscidin-1 induces cell death in HeLa and HT1080 cells with an IC_50_ of ~7–8 µM^28^, similar to that in our results. Furthermore, our experiments show that the antitumor activity of piscidin-1 in OSA cells was initiated by inducing apoptosis, and this finding is consistent with the results of a previous report^28^.

MtROS are harmful substances and are the undesirable byproducts of oxidative phosphorylation, which indefinitely damages cells; however, superoxide anion (O_2_^−^) and hydrogen peroxide (H_2_O_2_) are major ROS and important signaling molecules and are directly involved in the regulation of mitochondrial and cellular functions^39^. Considering that SOD2 enzymes can extensively catalyze undue ROS in the mitochondria, they are involved in ROS production and in changes in the respiratory chain complex process^40^. Apoptosis induction uses two main pathways, namely, extrinsic (death receptor) and intrinsic (mitochondria mediated)^41,42^. The intrinsic pathway regulates the permeability of the mitochondrial outer membrane and creates pores in this membrane that cause apoptosis-inducing factors, such as cyt *c*, to be constantly released into the cytoplasm. Cyt *c* and caspase-9 combine to form an apoptosome and subsequently activate caspase-3, thus causing apoptosis and death. It is worth noting that the accumulation of mtROS precedes MTP (ΔΨm) damage, nuclear condensation, and apoptotic body formation^43^. This study demonstrated that the effective cytotoxicity of and the apoptosis induced by piscidin-1 on OSA cells is achieved by the induction of mtROS and the disruption of MTP; however, we also observed that piscidin-1 increases mtROS levels at very low concentrations (0.1 µM) before the observed MTP dissipation (1 µM).

The mitochondria plays many important roles in eukaryotic cells, the most important of which is the production of ATP during the OXPHOS process^44^. There are two ways that cells produce ATP: OXPHOS (in the mitochondria) and non-mitochondrial (in the cytoplasm) processes. Our study showed that piscidin-1 reduces non-mitochondrial and OXPHOS respiration, including the basal OCR, ATP production, maximum respiration OCR, spare respiration capacity, and non-mitochondrial respiration but has no effect on proton leak respiration in OSA cells. The inner membrane of the mitochondria has many folds (cristae), which accommodate many copies of the respiratory chain component or OXPHOS complexes I–V. Complexes I–V is multi-subunit enzymes that synergistically produce an electrochemical proton gradient on the inner mitochondrial membrane. Finally, together with complex V (ATP synthase), they form the machinery for producing ATP, which is the source of cell metabolism and cell biosynthesis and is also known as the energy currency of cells^45^. The expression levels of complex I–V proteins were gradually reduced with increased concentrations of piscidin-1. The cell mitochondrial stress test using a Seahorse XF24 Analyzer and the expressions of complex I–V proteins indicated that the mitochondria exhibited a two-stage change as the piscidin-1 concentration increased. First, it was stimulated and damaged, efficiency was slightly reduced, and it maintained stable operations. Second, if the damage was too extensive, it would not be able to work appropriately, and the entire cell would be on the verge of collapse. This indicated that piscidin-1 has obvious apoptosis effects on OSA cells by inhibiting mitochondrial OXPHOS and the expression of complexes I–V, thus decreasing ATP production and finally causing mitochondrial dysfunction.

Mitochondria are dynamic organelles that exhibit fusion (combining pieces) and fission (splitting into smaller pieces) via the active recruitment of specific proteins to the indicated locations within the organelle. Mitochondrial fusion requires three large GTPases, the outer membrane proteins MFN1 and MFN2 and the inner membrane protein OPA1. The activation of GTPase DRP1 and the outer membrane protein FIS1 is necessary for mitochondrial fission. Mitochondrial fusion and fission have several important functions. They control the morphology of the mitochondria and allow for the exchange of content between the mitochondria, thus controlling mitochondrial distribution and promoting the release of membrane gap proteins during apoptosis^46^. Several structural changes in the mitochondria are important for rapid and efficient apoptosis they must have a fragmented expression with the permeable outer membrane, and the cristae must be disconnected; therefore, mitochondrial fission has significant implications in oxidative stress response and apoptosis^47^. Our results are consistent with the aforementioned observation, i.e., the induction of apoptosis by piscidin-1 is responsible for a decrease in the mitochondrial fusion proteins and an increase in the mitochondrial fission proteins in OSA cells.

MitoTempo is a mitochondria-targeted antioxidant with superoxide scavenging properties, i.e., it helps protect against oxidative damage to the mitochondria^48^. Many researches have proved that mitoTempo maintains mitochondrial integrity and reduces necrosis and apoptosis^48,49^. Our results demonstrated that piscidin-1 induces OSA cell apoptosis by increasing mtROS production and destroying MTP. Pretreatment with mitoTempo significantly decreases the overproduction of mitochondrial superoxide radical, the disruption of MTP, and the apoptosis mediated by perscidin-1 in the OSA cells.

Most of the defense peptides with anticancer activity are cationic and the molecular structures are either α-helical or β-sheet. Compared with normal cells, the cellular membrane of cancer cells contains more anions such as heparin sulfate and phospholipid phosphatidylserine, so that the cell membrane exhibits net negative charge. During the early stage of apoptosis, the mitochondria gradually become negatively charged with the increase of cardiolipin exposure; therefore, piscidin-1 entering OSA cells will attack and destroy the integrity of the mitochondria^50^. Current studies suggest that the selectivity and underlying mechanism of these anti-cancer peptides depends on invasion of mitochondrial and/or plasma membranes of cancer cells by potential differences, leading to cell apoptosis^51^. Piscidin-1 with positive charge possesses an α-helical structure, and the specific anticancer ability we observed may be caused by the aforementioned mechanism^28^.

**Figure 8:**
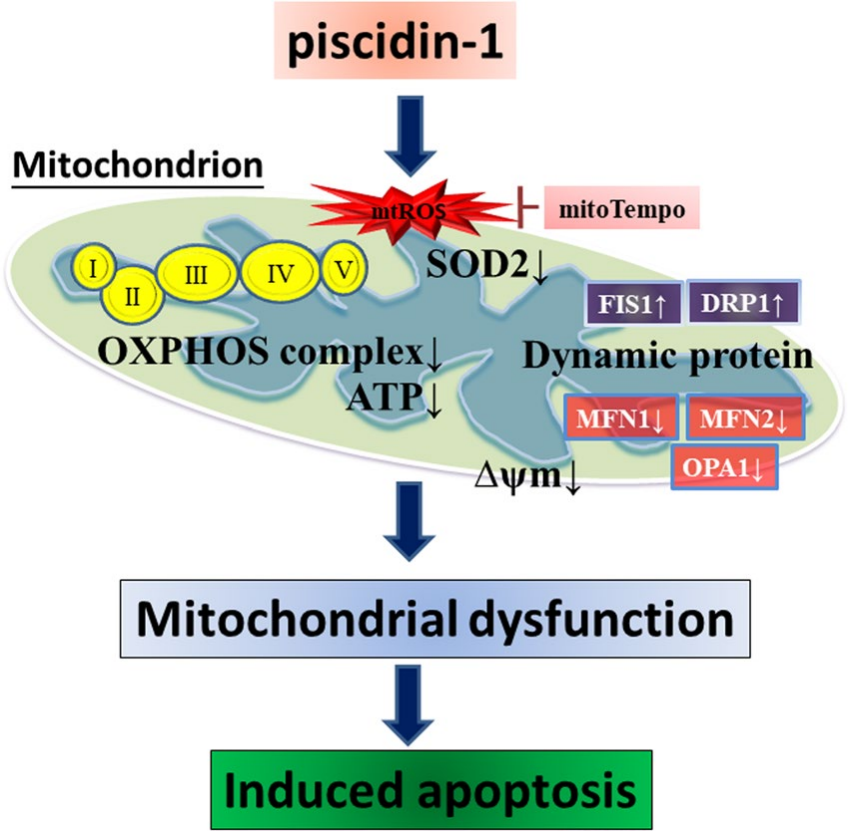
Overview of piscidin-1 anticancer signaling pathway. Piscidin-1 induces the formation of oxidative damage by accumulating mtROS, inhibiting SOD2, disrupting mitochondrial transmembrane potential (ΔΨm), depleting ATP metabolism, reducing OXPHOS complexes I–V, creating an imbalance in mitochondrial dynamic proteins and disorder in mitochondrial respiration. This leads to mitochondrial dysfunction and the formation of cancer cell apoptosis. Pretreatment with mitoTempo, mtROS scavenger, could rescue the apoptosis induced by piscidin-1.

The results of this study showed that piscidin-1, which is a marine pepide, inhibits MG63 and 143B OSA cell growth by inducing the apoptosis pathway (Fig. 8). Piscidin-1 treatment enhances mtROS production and decreases SOD2 antioxidant expression, followed by disrupting OXPHOS, decreasing the expression of complex I–V proteins, inducing an imbalance in mitochondrial dynamic proteins (including increased fission proteins and decreased fusion proteins), dissipating MTP (Δψm) and ATP production, and causing cancer cell apoptosis. MitoTempo is effective in reducing the apoptosis mediated by piscidin-1. These results indicate that piscidin-1 possesses anticancer effects by increasing mtROS and inducing mitochondrial dysfunction. To the best of our knowledge, this is the first report to demonstrate that piscidin-1 can inhibit human cancer growth by influencing mtROS, OXPHOS, and mitochondrial function. The above characteristics indicate that piscidin-1 has the potential for additional research and development as a new chemotherapeutic drug.

## Methods

### Cell lines and culture

MG63 (ATCC®CRL-1427) and 143B (ATCC®CRL-8303) osteosarcoma cells, lung cancer A549 cells (ATCC®CCL-185), ovarian cancer SKOV3 cells (ATCC®HTB-77), and primary gingival fibroblast (HGF-1) cells (ATCC®PCS-201-018) were purchased from the American Type Culture Collection (Manassas, VA, USA). Human oral mucosal fibroblast (OMF) cells were a kind gift from Professor Michael Hsiao of Academia Sinica Institute, Taiwan. A549 cells were maintained in F12K medium (Gibco BRL, Rockville, MD, USA), HGF-1 and OMF cells were cultured with Dulbecco’s Modified Eagle’s Medium (DMEM; Gibco), SKOV3 cells were cultured in Roswell Park Memorial Institute 1640 medium (Gibco), and MG63 and 143B cells were cultured with Minimum Essential Medium Eagle Alpha Modification medium (Gibco). Ten-percent heat-inactivated fetal calf serum was added to all cells (Invitrogen, Carlsbad, CA, USA), and the cells were incubated in penicillin/streptomycin in a 5% CO_2_ incubator at 37 °C. The attached cells had the morphology of cobblestone when reaching confluence.

### Compound and reagent

Piscidin-1, which is a 22-amino-acid peptide, was dissolved in PBS (pH = 7.2), protected from light, and stored at −20 °C. MTT for cell viability, annexin V–FITC/PI kit for apoptosis and cell death, MitoSOX Red for mtROS, and rhodamine 123 for MTP were purchased from Molecular Probes, Inc. (Eugene, OR, USA) and dissolved in DMSO. The TUNEL kit for the apoptosis experiment was purchased from Roche Applied Science (Mannheim, Germany). The Seahorse XF Cell Mito Stress Test was purchased from Agilent Technologies, Inc. (Santa Clara, CA, USA). The mitoTempo was obtained from Santa Cruz Biotechnology (Santa Cruz, CA, USA) and dissolved in DMSO.

### Scanning electron microscopy morphological analysis

The scanning electron microscopy sample was prepared on the basis of the methods of Kung *et al*.^52^. The cell cultures were washed with PBS and subjected to dehydration in a graded alcohol series for 2 min each (10%, 25%, 50%, 75%, and absolute ethanol v/v % ethanol). After dehydration, the samples were mounted on copper plates and sputter-coated with a thin layer of gold. Finally, the scanning electron microscope images were obtained using the JEOL JSM-7000F scanning electron microscope at an accelerating voltage of 5 kV.

### Cell viability

The cells were seeded at an initial density of 6 × 10^3^ cell/well in 96-well plates and placed on a culture plate into a CO_2_ incubator at 37 °C overnight. After piscidin-1 treatment with 0, 0.1, 1, 5, and 10 µM for 24, 48, and 72 h, 20 µL 5 mg/mL MTT solution was added into the cell-attached wells for 4 h. The supernatant was removed and intracellular formazan compounds were solubilized in 100 µL/well DMSO at room temperature for 15 min. The absorbance at a wavelength of 570 nm was measured using an enzyme-linked immunosorbent assay microplate reader (Dynatech Laboratories, Chantilly, VA, USA). The relative cell viability was calculated as the percentage of cells treated with piscidin-1 and those in the untreated control group.

### Flow cytometric analyses for apoptosis

Apoptosis was measure by fluorescence levels after staining with annexin V–FITC and PI. The seeded 5 × 10^5^ cells were plated in six-well plates and incubated overnight. MG63 and 143B cells were treated with piscidin-1 at 0, 0.1, 1, 5, and 10 μM for 24 h, another group was treated with or without mitoTempo for 2 h, and 10 μM piscidin-1 was added and allowed to react for 24 h. The resuspended cells at 1 × 10^5^ cells/100 μL in working binding buffer (HBSS) containing 5 μL purified recombinant annexin V–FITC and 5 μL PI were gently mixed and incubated for 10 min at room temperature to avoid light, and 1 mL HBSS solution was added. Beckman Coulter’s CytoFLEX (Southfield, MI, USA) was used to detect the fluorescence intensity of annexin V (green fluorescence)/PI (red fluorescence). At least 20,000 cells/group were analyzed using CytExpert 2.0 software (Beckman Coulter).

### TUNEL staining and fluorescence image

For the TUNEL assay purchased from Roche, 1 × 10^5^ cells/well were set on glass coverslips for each well in a 12-well plate for at least 24 h and were subsequently treated with piscidin-1 for 24 h. The cell culture medium was carefully removed and washed twice with PBS, and the MG63 cells were fixed and blocked with a 4% neutral formalin and 3% BSA solution for 15 min. The staining procedure followed the manufacturer’s instructions. The cell nuclei were stained with DAPI as a nuclear position. Fluorescence was visualized using the Leica TCS SP5 II confocal microscope (Wetzlar, Germany).

### Flow cytometric analyses for mtROS and MTP

The fluorescence levels of mtROS were measured after staining with 10 μM MitoSOX Red (red fluorescence) for 20 min at 37 °C. MTP was indicated using rhodamine 123 (green fluorescence) and flow cytometry in the model of FITC according to the manufacturer’s instructions. The cells were seeded at a density of 5 × 10^5^ cells/well in a six-well plate. After treatment with various concentrations of piscidin-1, mitoTempo, or cotreatment of piscidin-1 and mitoTempo, the fluorescent dyes were added at the proper volumes into the HBSS solution, and the solution was incubated at 37 °C for 20 min. Subsequently, the medium was removed, and trypsin was added to the cells. The cells were then resuspended in 1 mL HBSS. The CytoFLEX (Beckman Counter) was used to detect the fluorescence intensity of mtROS and MTP. At least 20,000 cells/group were analyzed using CytExpert 2.0 software (Beckman Coulter).

### Mitochondrial and cytosol isolation assay

The MG63 cells were treated with 0, 0.1, 1, 5, and 10 μM piscidin-1 for 24 h, and the mitochondria and cytosol were separated and isolated using the Mitochondria/Cytosol Fractionation kit (BioVision, Inc., Milpitas, CA, USA) according to the manufacturer’s instructions. Thereafter, Western blot analysis was conducted.

### Western blot analysis

The MG63 cells were seeded at a density of 1 × 10^6^ cells/well in a 6 cm dish and were pretreated with 0, 0.1, 1, 5, and 10 μM piscidin-1 for 24 h. RIPA buffer (Thermo Fisher Scientific, USA) was used on the cells to isolate total protein, and the lysates were centrifuged at 13,000 rpm at 4 °C for 30 min to obtain soluble proteins in the supernatant. A BCA assay was then used to determine protein concentrations (Bio-Rad, Hercules, CA, USA). Extracts of protein were taken from each group and then fractionated using 8–15% sodium dodecyl sulfate–polyacrylamide gel electrophoresis to separate the proteins. Proteins were transferred to a polyvinylidene difluoride (PVDF) membrane (Millipore, Bedford, MA, USA). Antibodies for cyt *c*, COX IV, SOD2, cleaved caspase-3 and cleaved caspase -9 (Cell Signaling Technology, Danvers, MA, USA), β-actin, mitochondrial complex protein (complexes I, II, III, IV, and V), and mitochondrial dynamic-related protein (MFN1, MFN2, OPA1, DRP1, and FIS1) (Abcam, Cambridge, UK) were used. The membranes were incubated at 37 °C for 1 h with horseradish peroxidase–conjugated secondary antibodies. The membrane was placed on a visualization strip by using a chemiluminescence kit (Millipore, Darmstadt, Germany) and UVP BioChemi imaging (UVP LLC, Upland, CA, USA). The PVDF membrane was reprobed using a β-actin antibody as a loading control. The relative densitometry of the bands was quantified using ImageJ, normalized with that of the β-actin and COX IV levels, and expressed as fold changes.

### ATP concentration assay

MG63 cells were seeded in triplicate at a density of 2 × 10^5^ cells/well in six-well plates and then treated with various concentrations of piscidin-1 for 24 h. The cells were harvested in a lysis buffer (20 mM glycine, 50 mM MgSO4, and 4 mM ethylenediaminetetraacetic acid)^53^. ATP was measured using the ATP Colorimetric/Fluorometric Assay kit (BioVision, Inc., Milpitas, CA, USA) according to the manufacturer’s instructions. Twenty microliters from each sample were mixed with 4 μL ATP solution for fluorescence readings at Ex/Em 535/587 nm by using a fluorescence meter. The amount of ATP production was determined from a standard curve constructed with 10–100 pmol ATP.

### Live-cell metabolic assay

OCR was measured using the XF24 Seahorse XF Analyzer (Seahorse Bioscience, North Billerica, MA, USA). MG63 and 143B cells (30,000/well) were cultured onto XF24 polystyrene cell culture plates and then treated with piscidin-1 for 24 h. The old medium was removed, and 675 μL of DMEM medium that was adjusted to pH 7.4 without sodium bicarbonate was added before using the XF24 Analyzer. For three injection stages, 1 μM oligomycin, 0.5 μM FCCP, and 1 μM antimycin A/rotenone were sequentially added into the assay medium. The attached cells were lysed using RIPA, and a BCA kit was used to measure the optical density at 570 nm to normalize OCR value.

### Statistical analyses

SPSS ver. 17 (SPSS Inc., Chicago, IL, USA) was used to analyze the data. The variables analyzed using the independent Student’s *t-*test were expressed as mean ± standard error. *p < 0.05 and **p < 0.01 were considered statistically significant analyses.

## Supplementary information

Supplementary Information.

## Data Availability

All data generated or analyzed during this study are included in the published article (and its Supplementary Information file).
