# Supplementary material for: Piscidin-1 Induces Apoptosis via Mitochondrial Reactive Oxygen Species-Regulated Mitochondrial Dysfunction in Human Osteosarcoma Cells
Source: Sci Rep. 2020 Mar 19;10:5045. doi: 10.1038/s41598-020-61876-5 (PMC7081333; doi:10.1038/s41598-020-61876-5)
Supplement: Supplementary file 1 — Supplementary Information. [file 41598_2020_61876_MOESM1_ESM.pdf]

Supplementary Information

Piscidin-1 Induces Apoptosis via Mitochondrial Reactive Oxygen Species-Regulated Mitochondrial Dysfunction in Human Osteosarcoma Cells

Meng-Hsuan Cheng[1,2,3], Chieh-Yu Pan[4], Nan-Fu Chen[5,6], San-Nan Yang[7], Shuchen Hsieh[8], Zhi-Hong Wen[9,10], Wu-Fu Chen[9,11,12], Jin-Wei Wang[13]*, Wen-Hsien Lu[14], Hsiao-Mei Kuo[9,15]*****

[1] Division of Pulmonary and Critical Care Medicine, Department of Internal Medicine, Kaohsiung Medical University Hospital, Kaohsiung, 80756, Taiwan; cmhkmu@gmail.com (M.-H.C.)

[2] School of Medicine, College of Medicine, Kaohsiung Medical University, Kaohsiung, 80708, Taiwan; cmhkmu@gmail.com (M.-H.C.)

[3] Department of Respiratory Therapy, College of Medicine, Kaohsiung Medical University, Kaohsiung, 80708, Taiwan; cmhkmu@gmail.com (M.-H.C.)

[4] Department and Graduate Institute of Aquaculture, National Kaohsiung University of Science and Technology, Kaohsiung 81101, Taiwan; panjade@webmail.nkmu.edu.tw (C.-Y.P)

[5] Division of Neurosurgery, Department of Surgery, Kaohsiung Armed Forces General Hospital, Kaohsiung, 80284, Taiwan; chen06688@gmail.com(N.-F.C.)

[6] Department of Neurological Surgery, Tri-Service General Hospital, National Defense Medical Center, Taipei, 11490, Taiwan; chen06688@gmail.com(N.-F.C.)

[7] Department of Internal Medicine, E-DA Hospital and College of Medicine, I-SHOU University, Kaohsiung, 84001, Taiwan; y520729@gmail.com(S.-N.Y.)

[8] Department of Chemistry, National Sun Yat-sen University, Kaohsiung, 80424, Taiwan; shsieh@faculty.nsysu.edu.tw (S.-C.H)

[9] Department of Marine Biotechnology and Resources, National Sun Yat-sen University, Kaohsiung, 80424, Taiwan; wzh@mail.nsysu.edu.tw (Z.-H.W.); ma4949@cgmh.org.tw(W.-F.C)

[10] Doctoral Degree Program in Marine Biotechnology, Academia Sinica, Taipei, 11529, Taiwan; wzh@mail.nsysu.edu.tw (Z.-H.W.)

[11] Department of Neurosurgery, Kaohsiung Chang Gung Memorial Hospital and Chang Gung University College of Medicine, Kaohsiung, 83301, Taiwan; ma4949@cgmh.org.tw(W.-F.C)

[12] Department of Neurosurgery, Xiamen Chang Gung Hospital, Xiamen, Fujian, China; ma4949@cgmh.org.tw(W.-F.C)

[13] Department of Orthopedic, Kaohsiung Armed Forces General Hospital, Kaohsiung, 80284, Taiwan; xenoprophet@gmail.com(J.-W.W)

[14] Department of Orthopedic, Feng Yuan Hospital of the Ministry of Health, Taichung, 42055, Taiwan; 97jack@gmail.com(W.-H. L.)

[15] Center for Neuroscience, National Sun Yat-sen University, Kaohsiung, 80424, Taiwan; hsiaomeikuo@gmail.com(H.-M.K.)

*****Correspondence to:** Hsiao-Mei, Kuo Ph.D. Center for Neuroscience and Department of Marine Biotechnology and Resources, National Sun Yat-sen University, #70 Lien-Hai Rd, Kaohsiung, 80424, Taiwan. E-mail: Hsiaomeikuo@gmail.com.

TEL: +886-7-5252000 #5038

FAX: +886-7-5252021

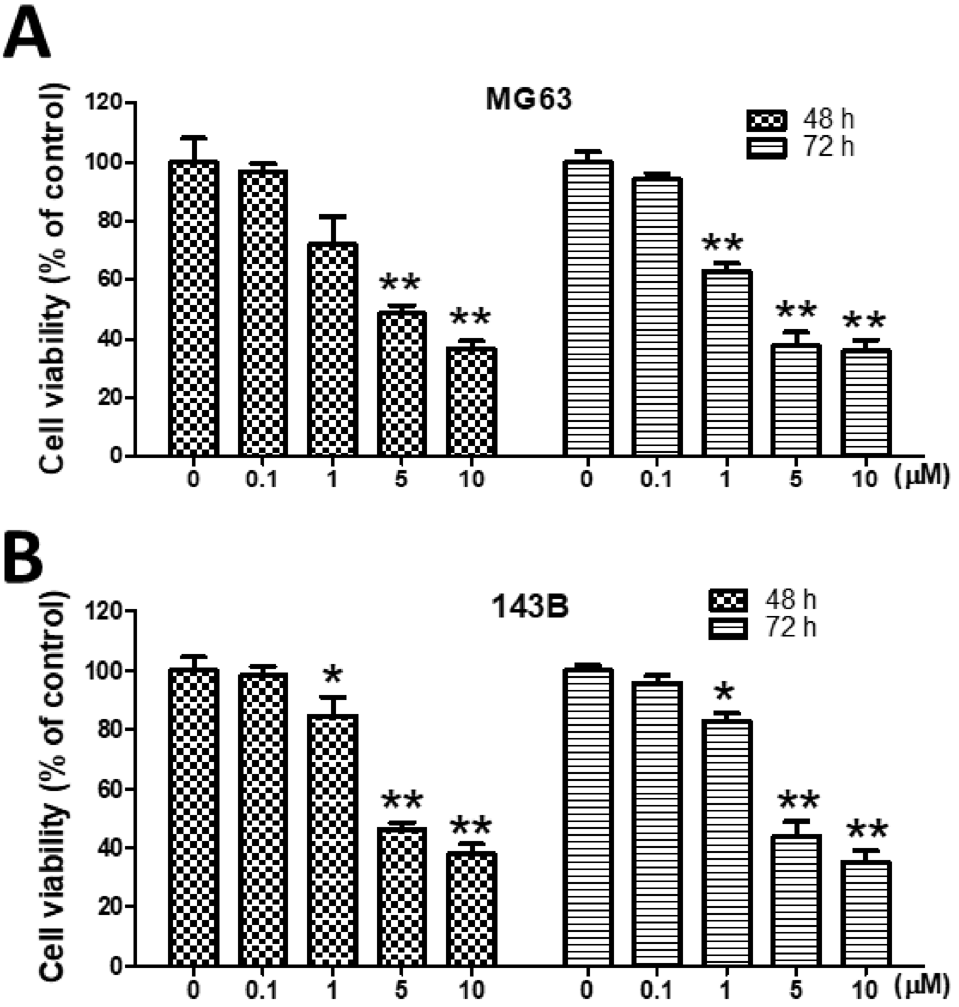

Figure S1. Effect of piscidin-1 on the cell viability in MG63 and 143B cells. (A) Human OSA MG63 and 143B cells were treated with the 0, 0.1, 1, 5, and 10 μM piscidin-1 for 48, 72 h, and then, the MTT assay was performed to measure cell viability. The cell viability of (A) MG63 and (B) 143B cells decreased along with increasing concentrations of piscidin-1. Cell viability is expressed as a percentage of the untreated control cells (0 μM). The results are expressed as the mean ± SEM of three independent experiments. The results are expressed as the mean ± SEM of three independent experiments. Significance was determined using Student's *t*-test; *$p < 0.05$; **$p < 0.01$.

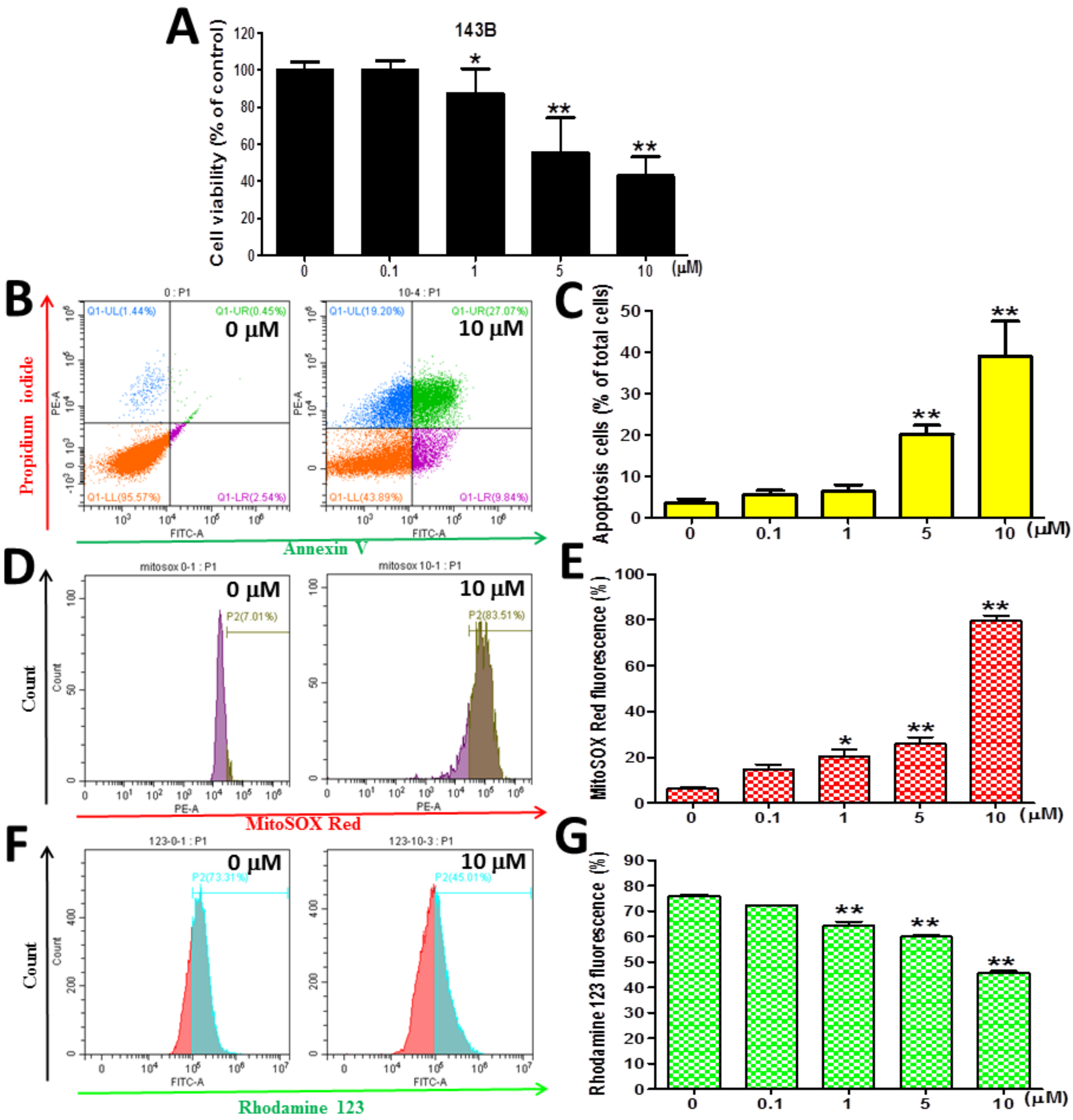

Figure S2. Effect of piscidin-1 on the cell viability, apoptosis, mtROS production, and MTP dissipation in 143B cells. (A) Human OSA 143B cells were treated with the 0, 0.1, 1, 5, and 10 μM piscidin-1 for 24 h, and then, the MTT assay was performed to measure cell viability. The cell viability of 143B cells decreased along with increasing concentrations of piscidin-1. Cell viability is expressed as a percentage of the untreated control cells (0 μM). The results are expressed as the mean ± SEM of three independent experiments. (B) Apoptosis was determined using annexin V–FITC/PI staining and flow cytometry of the 143B cells treated with or without piscidin-1 (10 μM) for 24 h. The dot-plot quadrant diagram reflects the annexin V–FITC (x-axis; green) and PI (y-axis; red) in the 143B cells. (C) The percentages of apoptotic cells (lower right quadrant) and dead cells (upper right quadrant) in 143B cells treated with the 0, 0.1, 1, 5, and 10 μM piscidin-1 for 24 h were examined by flow cytometry analysis. The apoptotic 143B cells increased as the concentrations of piscidin-1 increased. Total cells = 20,000; values

are the mean ± SEM of three independent experiments. (D) The fluorescent intensity of mtROS was determined using Mitochondrial Superoxide Indicator (MitoSOX Red) (red fluorescence) and was detected by flow cytometry in the 143B cells treated with or without piscidin-1(10 μM) for 24 h. Mitochondrial ROS accumulation was enhanced after treatment with 10 μM piscidin-1 for 24 h, and flow cytometry showed a considerable shift to the right in 143B cells. (E) The quantitative results indicated that at concentrations of 1, 5, and 10 μM piscidin-1, mtROS in 143B cells clearly increased; data was analyzed by Beckman CytoFLEX flow software. Values are the mean ± SEM of three independent experiments. (F) The fluorescent intensity of MTP depolarization was determined using Rhodamine 123 dye (green fluorescence) and was detected by flow cytometry in the 143B cells treated with or without piscidin-1(10 μM) for 24 h. In the 143B cells treated with 10 μM piscidin-1 for 24 h, MTP was disrupted, and the flow cytometry histogram showed a considerable shift to the left. (G) Percentages of MTP-disrupted 143B cells increased with increasing concentrations of piscidin-1. The quantitative value was obtained by analyzing the gated range of single-parameter histograms from 10^4–10^7. The results are expressed as the mean ± SEM of three independent experiments. Significance was determined using Student's *t*-test; $*p < 0.05$; $**p < 0.01$.

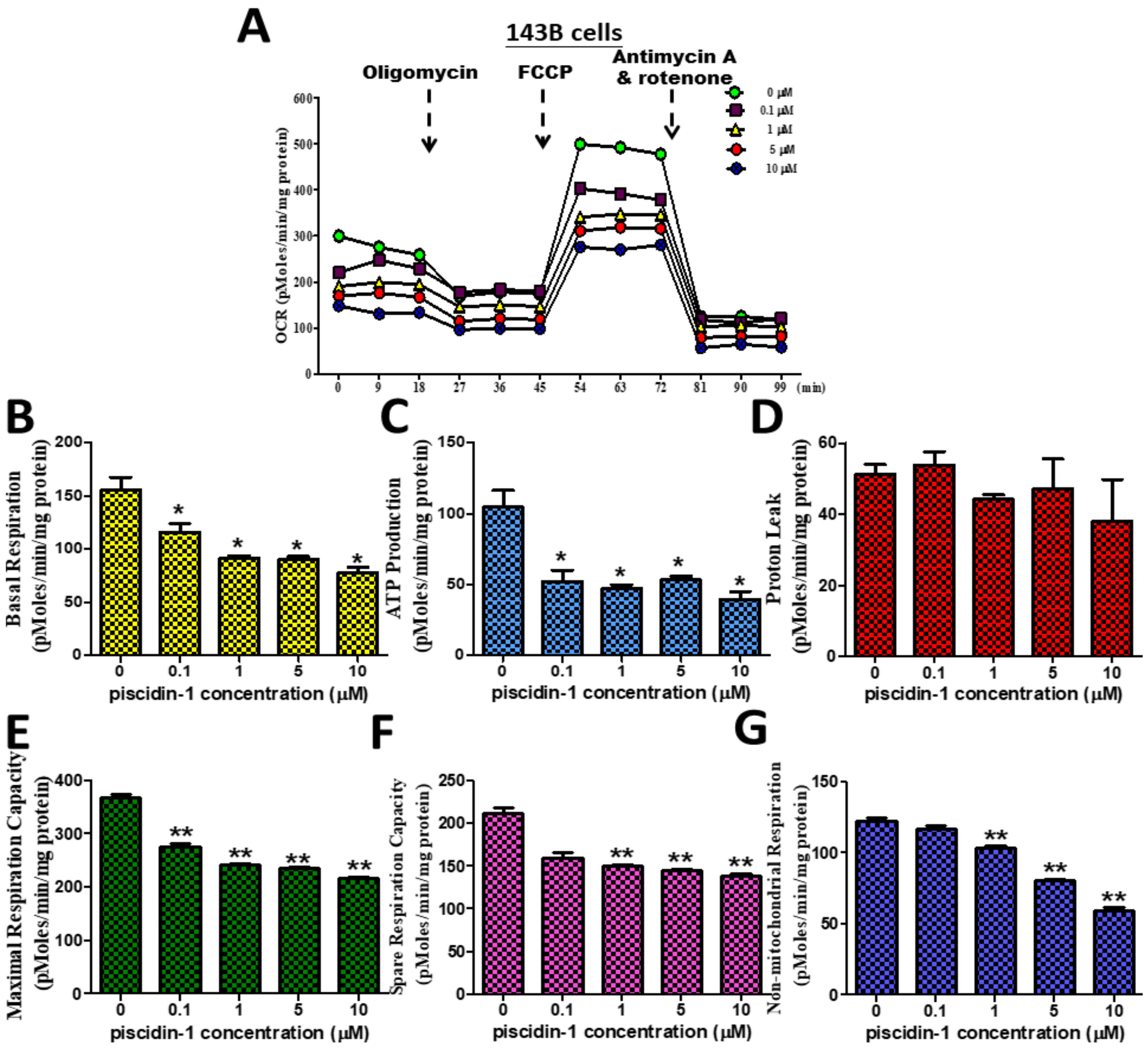

Figure S3. The effect of piscidin-1 on mitochondrial function and respiration in the 143B cell line. The high-throughput Seahorse XF24 apparatus was used to examine changes of mitochondrial function and oxygen consumption after piscidin-1 treatment. (A) The OCR value and time course curve plot. OCR in basal and different concentrations (0.1, 1, 5, and 10 μM) of piscidin-1 treatment for 24 h in 143B cells, followed by sequential and continuous injections of oligomycin, FCCP, and antimycin A/rotenone. These analyses measured the basic parameters of basal respiration, ATP production, proton leak, maximum respiration, spare respiratory capacity, and non-mitochondrial respiration. The parameters quantified and analyzed in 143B cells treated with various doses of piscidin-1 were the (B) basal respiration OCR, (C) ATP production OCR (couple respiration), (D) proton leak respiration OCR, (E) maximal respiration OCR, (F) spare respiratory capacity OCR, and (G) non-mitochondrial respiration OCR. OCR levels were quantified by normalizing the cell protein concentration. The results are expressed as the mean ± SEM of three independent experiments. Significance was determined by Student's t-test; * $p < 0.05$; ** $p < 0.01$.

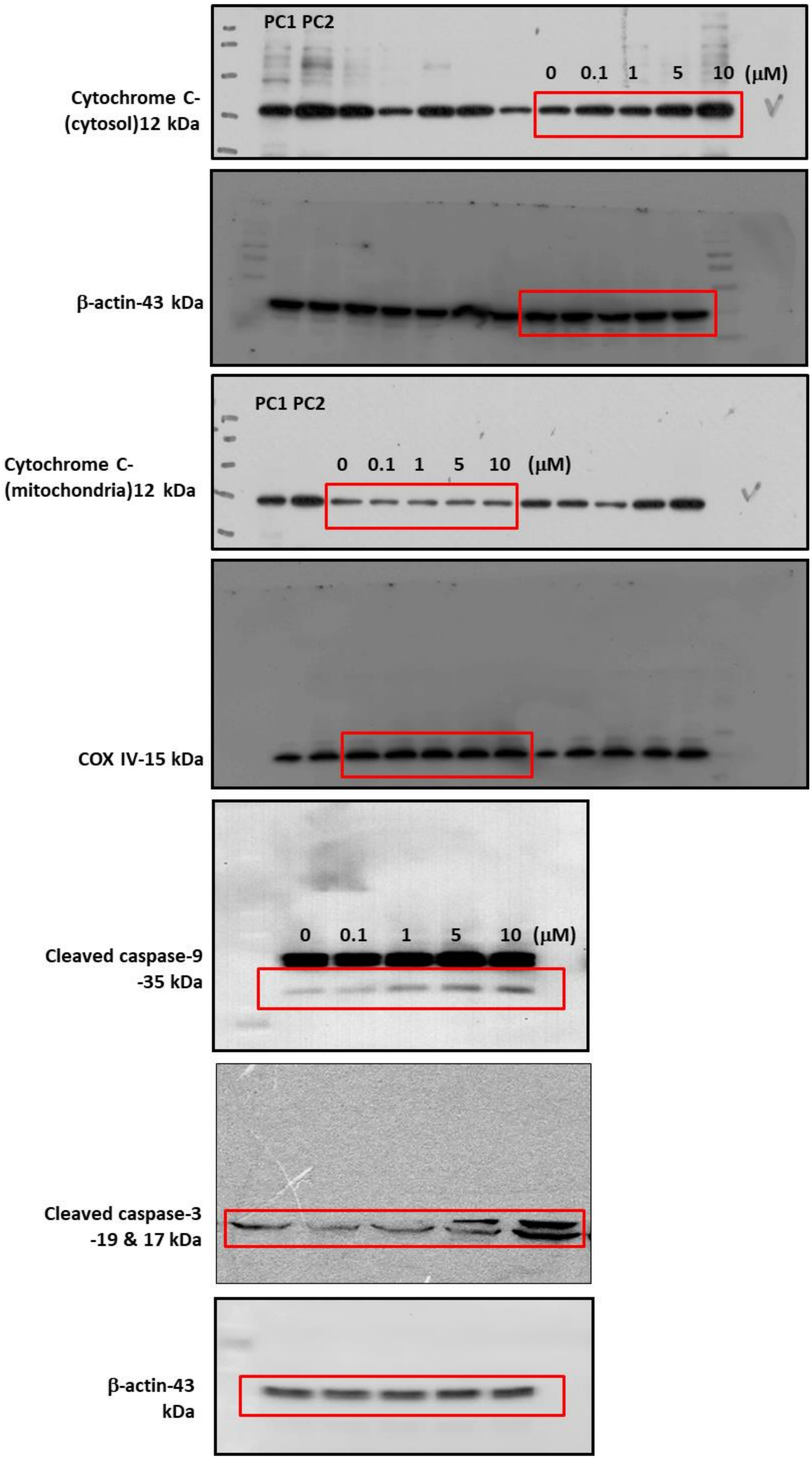

Figure S4. The figure showed the full original and uncropped images for the western blots of Fig. 2E displayed in the text and results. The identification of the

Cytochrome C, Cleaved caspase-9, Cleaved caspase-3, COX IV and β-actin bands was based on the expected molecular weight. The β-actin and COX IV were used as the cytosolic and mitochondrial protein loading control, respectively. (PC1: positive control 1; PC2: positive control 2)

SOD2-23 kDa

β-actin-43 kDa

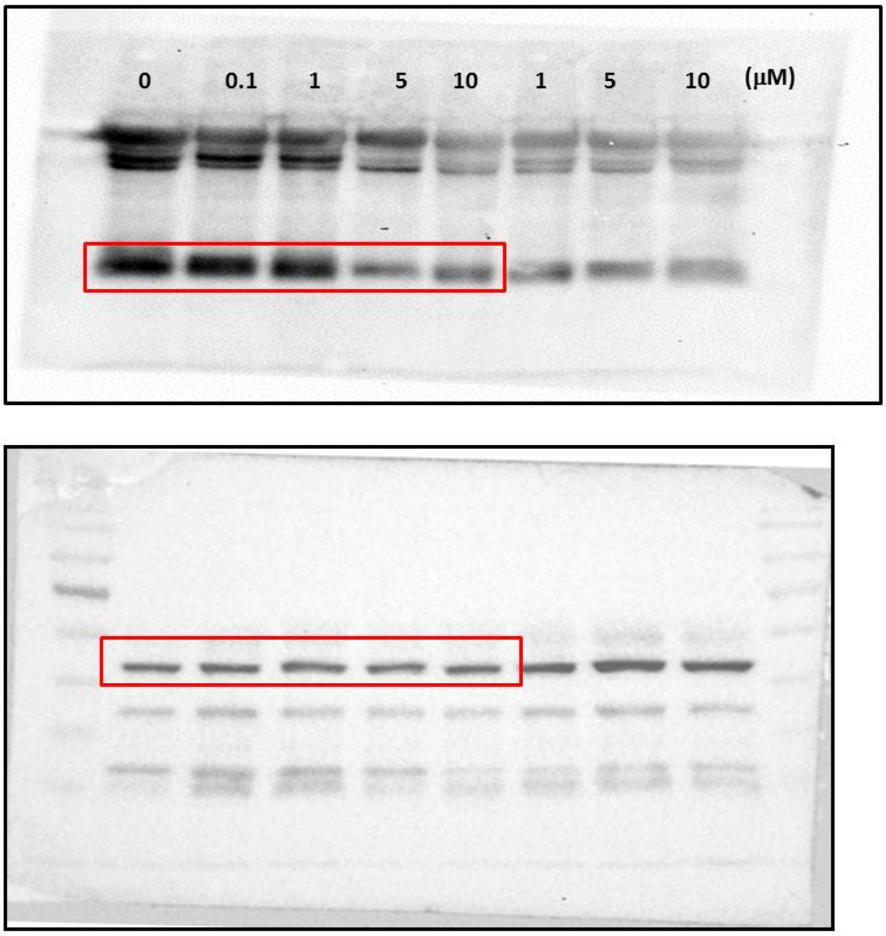

Figure S5. The figure showed the full uncropped blots of Fig. 3E displayed in the text and results. The identification of SOD2 and β-actin bands was based on the expected molecular weight. The β-actin was used as the protein loading control.

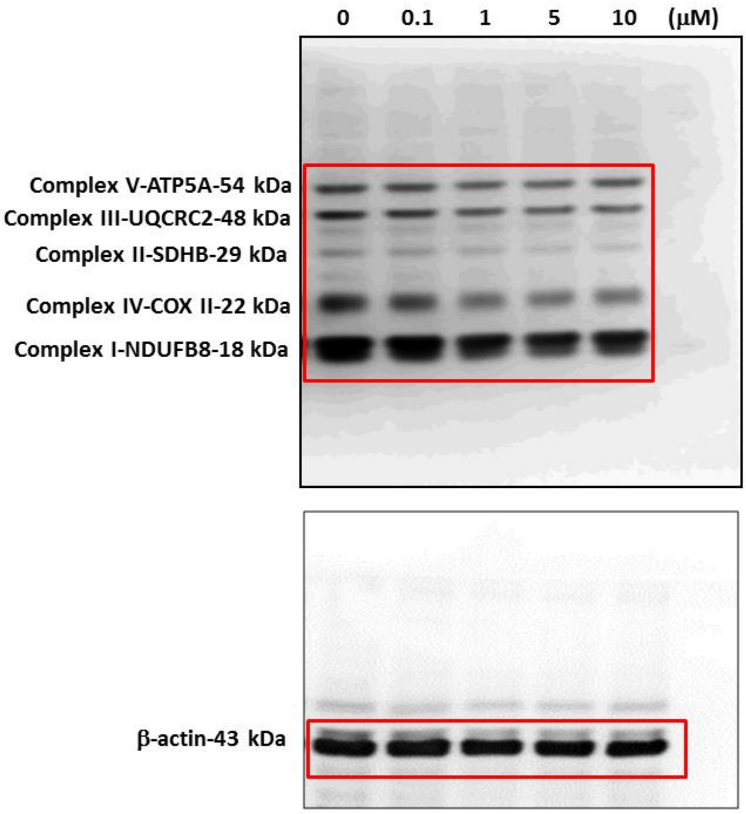

Figure S6. The figure showed the full original and uncropped images for the western blots of Fig. 5A displayed in the text and results. The identification of indicated protein expression was examined by Total OXPHOS Human WB Antibody Cocktail (Abcam, Cambridge, UK) based on the expected molecular weight. The β-actin was used as the protein loading control.

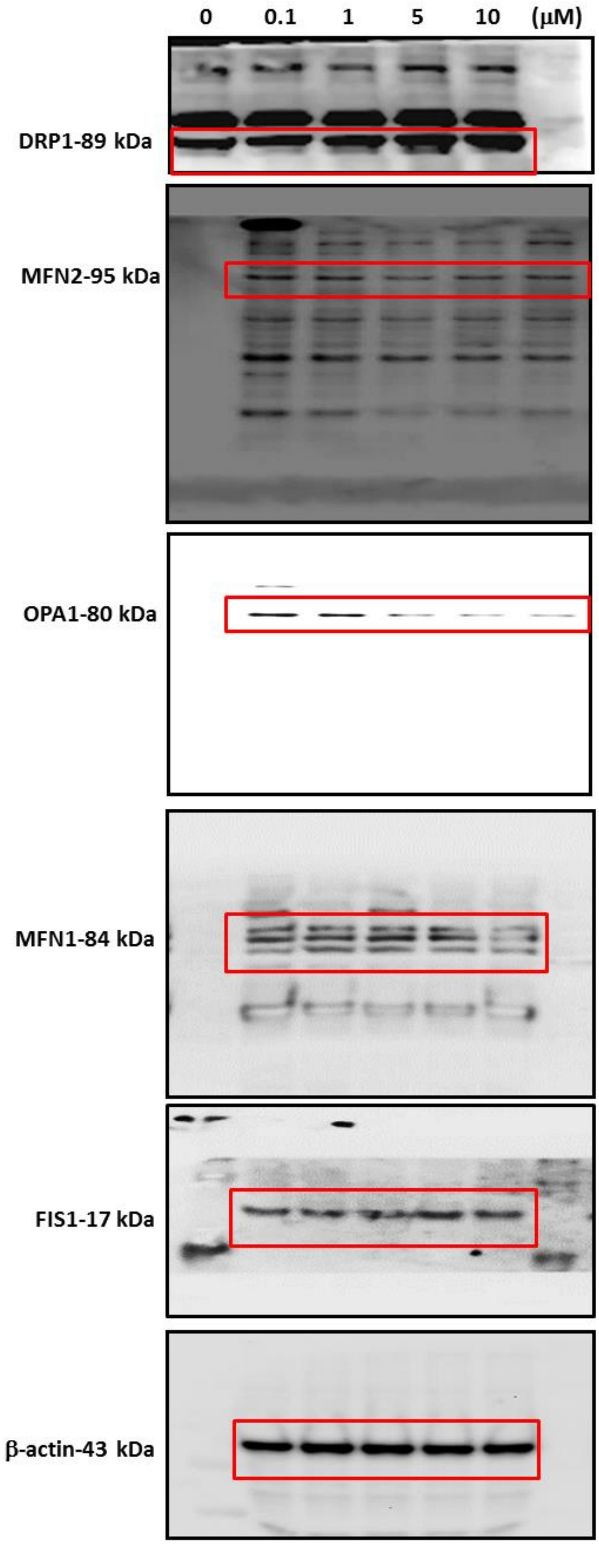

Figure S7. The figure showed the full original and uncropped images for the western blots of Fig. 6A displayed in the text and results. The identification of MFN1, MFN2, OPA1, FIS1, DRP1, and β-actin bands was based on the expected molecular weight. The β-actin was used as the protein loading control.
